# Variant Klinefelter Syndrome With Xq Trisomy (47,X,i(X)(q10),Y): A Case Report and Review of the Literature

**DOI:** 10.7759/cureus.77351

**Published:** 2025-01-12

**Authors:** Jagadeeshwar Ghatanatti, Somprakash Dhangar, Babu Rao Vundinti

**Affiliations:** 1 Cytogenetics, Indian Council of Medical Research-National Institute of Immunohaematology, Mumbai, IND

**Keywords:** infertility, normal intelligence, normal stature, trisomy xq, variant klinefelter syndrome

## Abstract

Variant Klinefelter syndrome (VKS) presents with variable phenotypes, caused by the involvement of different genetic abnormalities leading to delays in confirming diagnosis and, therefore, delays in early management of this disorder. We describe a male with VKS and trisomy Xq resulting from an isochromosome X(i(Xq10)). He had characteristics of classical KS such as azoospermia, hypergonadotropic hypogonadism with elevated follicle-stimulating hormone (FSH) and luteinizing hormone (LH) levels, and bilateral atrophic testes. He achieved a normal intelligence level. However, we observed a normal stature appropriate for his height. There was no history of developmental delays or learning problems during childhood. His karyotype was found to be 47,X,i(X)(q10),Y, which indicates that extra copies of the long arm of Xq (trisomy Xq) have phenotypic expression. In conclusion, Trisomy Xq does not affect stature and neurological development and does not express classical phenotypes during early childhood. Hence, the cytogenetic screening of sex chromosomal abnormalities is important in cases of hypergonadotropic hypogonadism and testicle abnormality, which can help in appropriate genetic counseling.

## Introduction

The classical form of Klinefelter syndrome (KS), characterized by a 47,XXY karyotype, typically presents with phenotypes such as hypogonadism, small testes, tall stature, azoospermia, gynecomastia, and diminished cognitive abilities [1]. However, several variants of KS have been identified, including 47,XX,der(Y), 48,XXXY, 48,XXYY, and 49,XXXXY [2]. Structural abnormalities are rare among these variants and constitute approximately 0.3% of all KS cases [3]. These differences are mainly due to the involvement of different genetic mechanisms, such as chromosomal non-disjunction, leading to the formation of different genotypes. The resulting phenotype also varies from case to case and always presents a challenge for clinicians in making a confirmed diagnosis [4]. For instance, variant KS cases often present with a variable clinical phenotype, including some of the classical features of KS. This phenotype generally depends on the gene dosage, such as an extra copy of a complete or partial chromosome, mosaicism, or other chromosomal rearrangements, which lead to a clinical presentation that is less stereotypical, with only some of the classic features of KS, such as infertility, hypogonadism, and small testes. Similarly, extra gene dosage of the Short stature homeobox-containing gene (SHOX), which is located on the p-arm of the X chromosome, can cause tall stature in these patients. Further, other risk factors such as increased maternal age and reduced recombination also found to play an important role in the development of human chromosomal aneuploidy [5]. Hence genotype-phenotype correlation becomes essential in individuals carrying such abnormalities for proper diagnosis and management. Here, we present a case of an infertile man with a rare variant of KS characterized by the presence of an isochromosome formed from the long arm (q arm) of one of the X chromosomes.

## Case presentation

A forty-year-old infertile man was referred for a cytogenetic study. On clinical examination, we observed decreased libido (low sex drive), erectile dysfunction, fatigue, and mood changes in this patient. His physical examination revealed bilateral, pea-sized testes with no other prominent features. According to his developmental history, he reached growth milestones and attained a normal level of intelligence. There was no history of delayed development or learning difficulties in early childhood. He achieved the expected height as per the mid-parental height (MPH) calculation and had a normal stature. His height and weight were 165 cm and 70 kg, respectively. Endocrinological evaluation through biochemical testing revealed high follicle-stimulating hormone (FSH) and luteinizing hormone (LH), with values of 69.04 mIU/mL (Reference range: 2.5-10 mIU/mL) and 34 mIU/mL (Reference range: 2.5-10 mIU/mL), respectively. His serum prolactin was found to be elevated and it was 26.48 mIU/mL (Reference range: 2.64-13.13 mIU/mL). His testosterone level was found to be nearly normal and it was 2.31 mIU/mL (Reference range: 2.80-8.00 mIU/mL). He also had a normal thyroid profile and cortisol level. The ultrasonographic examination of the testes showed no abnormalities in the epididymis or vas deferens, and there were no signs of varicocele. The karyotype of both parents was normal. There was no history of consanguinity and infertility in his family. 

Cytogenetics

**Figure 1:**
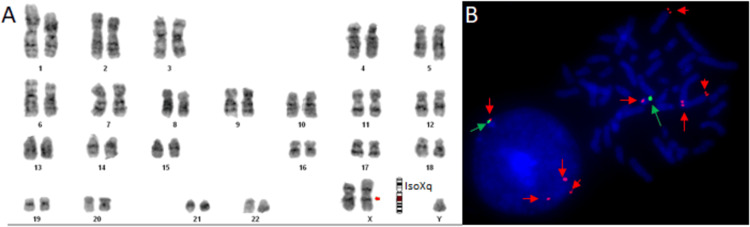
Cytogenetic analysis A. G-banded metaphase analysis showed a 47,X,i(X)(q10),Y karyotype. B. Fluorescently labeled DNA probes hybridized to metaphase and interphase cells revealed the presence of four red signals (denoted by red arrows) for the VAMP7 gene (350 kb), located on the PAR2 region of the long arm of the X chromosome and the q arm of the Y chromosome, indicating partial trisomy of Xq28. Additionally, one green signal (denoted by a green arrow) for the DYZ3 region, located near the centromere of the Y chromosome, indicates the presence of the Y chromosome. PAR: pseudoautosomal region

Blood lymphocyte cultures were performed according to standard procedures reported by Moorhead et al. [6]. Briefly, the blood cultures were activated with 300 uL phytohemagglutinin (Cat. no.10576-015, Gibco, USA ), halted using colchicine (50 µg/mL), and exposed to a hypotonic solution (Potassium chloride, 0.56 g/100 mL). The cells were subsequently preserved in Carnoy’s solution. The chromosomal samples obtained underwent Giemsa-Tripsin-Giemsa staining [7]. Karyotyping was performed (30 metaphases per case) at high resolution (300-400) according to universally accepted nomenclature protocol (ISCN 2020). Metaphase images were captured using a monochromatic camera connected to a fluorescent microscope (Nikon; Model-90i) and analyzed with Applied Spectral Imaging (ASI) software version 6.0 (ASITM, 6160 Innovation Way Carlsbad, CA, USA). Karyotyping analysis consistently revealed 47,X,i(X)(q10),Y karyotypes in all examined metaphases (Figure 1A).

Molecular cytogenetics

Fluorescence in situ hybridization (FISH) was conducted using a locus-specific dual-color DNA probe, following standard molecular cytogenetics protocols [8]. Briefly, chromosomal preparation and DNA FISH probe (Cat No. CT-PAC292; Cytotest, USA) were co-denatured and kept at 37°C in a humidified chamber for hybridization for 24 hours. After overnight hybridization, the slides were washed with washing solutions and stained with DAPI counterstain. The slides were mounted with mounting solution (Vectashield) and analyzed under a Nikon microscope (Model-90i; Nikon, Japan) attached to a CCD camera. The first probe, labeled in red, was specific to the VAMP7 gene (350 kb), located in the pseudoautosomal region 2 (PAR2) on the long arm of chromosome X (q28) and chromosome Y (q12). The second probe, labeled in green, targeted the DYZ3 region on chromosome Y. FISH analysis revealed the presence of four red signals for the VAMP7 gene (350 kb) located on the PAR2 region of both chromosomes. Three red signals were observed on chromosome X (q28), indicating trisomy Xq. Additionally, one red signal was detected on chromosome Y (q12) for the VAMP7 (350 kb) gene of the PAR2 region, and one green signal was observed for the DYZ3 region located near the centromere of chromosome Y (Figure 1B). The karyotype was confirmed as 47,X,i(X)(q10),Y and revealed partial trisomy for the Xq region. Therefore, no additional genetic testing was performed.

## Discussion

Classical KS is the commonest sex chromosomal disorder in humans, which affects spermatogenesis leading to infertility and other developmental anomalies such as gynecomastia, mental retardation, and abnormal tall stature. In contrast, variant KS cases always present with variable clinical presentation along with infertility; these phenotypes generally depend on the gene dosage present on the extra chromosomal material [2,9]. Classical KS is mostly diagnosed during adolescence age and the most noticeable phenotypic changes observed include delayed or incomplete puberty, reduced muscle mass and strength, gynecomastia (breast tissue development), and taller-than-average height with long limbs. Additional genetic material on the X chromosomes causes testicular tissue hardening and scarring, leading to reproductive abnormalities, typically gonadal insufficiency and sterility. The 47,X,i(X)(q10),Y karyotype found in this case reflects a rare variant of KS, with an anticipated prevalence of roughly one in 250 cases [10-14]. As access and expertise are increasing in the area of genetic technology day by day, our extensive literature search has revealed that only 25 cases of isochromosome Xq are described well in the literature to date (Table 1).

**Table 1:** Comparative clinical features and karyotype of 25 reported cases along with present case - Denotes not available. LH: luteinizing hormone; FSH: follicle-stimulating hormone

Case No.	Age	Height (cm)	Weight (Kg)	Gynaecomastia	Intelligence	LH	FSH	Testosterone	Karyotype	Reference
1	44	176	84	Yes	Normal	−	−	−	47,X,i(X)(q10),Y	[9]
2	36	164.5	−	No	Normal	Increased	Increased	Decreased	47,X,i(X)(q10),Y	[10]
3	29	163	53	−	−	−	−	−	47,X,i(X)(q10),Y	
4	24	166.4	78	No	Normal	Increased	Increased	Decreased	47,X,i(X)(q10),Y	
5	32	NR	NR	Yes	-	Increased	Increased	Decreased	47,X,i(X)(q10),Y	
6	33	168	63	Yes	Normal	Increased	Increased	Decreased	47,X,i(X)(q10),Y	
7	31	166	57	No	Normal	−	Increased	−	47,X,i(X)(q10),Y	
8	17	160	60	Yes	Normal	Increased	Increased	Decreased	47,X,i(X)(q10),Y	
9	30	165	59	No		Increased	Increased	Normal	47,X,i(X)(q10),Y	
10	28	198	89	−	−	−	Increased	Decreased	47,X,i(X)(q10),Y	
11	30	165	59	No	Normal	Increased	Increased	Normal	47,X,i(X)(q10),Y	
12	27	178	75	No	Normal	Increased	Increased	Normal	47,X,i(X)(q10),Y	
13	NR	NR	Prenatal	-	-	-	-	-	47,X,i(X)(q10),Y	
14	NR	NR	Prenatal	-	-	-	-	-	47,X,i(X)(q10),Y	
15	28	175	75	Yes	Normal	Increased	Increased	Decreased	47,X,i(X)(q10),Y	
16	21	171	66	No	Normal	Increased	Increased	Normal	47,X,i(X)(q10),Y	
17	41	170.5	77	No	Normal	Increased	Increased	Decreased	47,X,i(X)(q10),Y	
18	37	181	64	No	Normal	Increased	Increased	Decreased	47,X,i(X)(q10),Y	
19	40	168	−	No	Normal	Increased	Increased	Normal	47,X,i(X)(q10),Y	
20	30	174.5	91	No	Normal	Normal	Increased	Normal	47,X,i(X)(q10),Y	
21	37	170	63	No	Normal	Increased	Increased	Normal	47,X,i(X)(q10),Y	
22	36	180	91	No	Normal	Increased	Increased	Normal	47,X,i(X)(q10),Y	[11]
23	25	178	75	−	Normal	Increased	Normal	Normal	47,X,i(X)(q10),Y	[12]
24	33	160	53	No	Normal	Increased	Increased	Decreased	47,X,i(X)(q10),Y	[13]
25	32	163	78.5	No	Normal	Increased	Increased	Normal	47,X,i(X)(q10),Y	[14]
26	40	165	70	No	Normal	Increased	Increased	Normal	47,X,i(X)(q10),Y	Present case

According to these reports, the primary complaints among all patients with 47,X,i(X)(q10),Y karyotypes were infertility and azoospermia. While, all 25 reported cases of 47,X,i(X)(q10),Y was presented with normal intelligence. In comparison to prototypic KS patients, the noted differences included near-normal stature and no mental impairment in all assessed subjects, with the exception of one case reported by Kleczkowska et al. [15].

In our case, we have observed small testicular size and normal stature. These features are common among all previously reported cases with the 47,X,i(X)(q10),Y variants. The additional dosage of the SHOX gene present in the PAR site is responsible for the acromegalic phenotype [16]. However, the case with iso Xq (10) region does not contain the SHOX gene; hence they do not express phenotype related to tall stature and abnormal segment ratio. Therefore, the normal stature observed in patients with the karyotype 47,X,i(X)(q10),Y may be associated with the lack of overexpression of genes situated in the pseudoautosomal Xp region close to the telomere. The presence of additional features in cases of variant KS, such as eunuchoid habitus, sparse body hair, and the presence or absence of gynecomastia is highly variable. Previous studies on patients with morphologically altered X chromosomes have shown that extra material of the Xq site causes a lack of sperm in males and no sperm isolation from 47,X,i(X)(q10),Y patients have been reported in the literature [4,16]. These findings are consistent with the classical KS (47,XXY) phenotype. Recent studies also suggest that abnormal spermatogenesis in these patients could also be caused by defects in germ cells caused by X chromosome inactivation [17].

In 70%-90% of cases, classical KS occurs due to errors in either the first or second division of meiosis, resulting in non-disjunction and leading to aneuploidy of the X chromosome. In the remaining cases, non-disjunction occurs due to post-zygotic mitotic errors [18]. It is important to note that the isochromosome occurs due to the horizontal separation of chromosomes, which is rarely seen in humans. The cells containing an isochromosome exhibit trisomy for the duplicated arm while showing monosomy for the absent arm, resulting in varied phenotypic expression [3]. Gonadal insufficiency and sterility are observed in patients with both the 47,XXY karyotype and the 47,X,i(X)(q10),Y karyotype in KS. However, the severity of the phenotype increases with a higher number of X chromosomes, whereas individuals with fewer or partial segment duplications (on the p or q arm) tend to exhibit either mild phenotypes or remain clinically silent until puberty. According to the literature, only a small percentage (15%-30%) of classical KS cases are diagnosed before puberty, with the majority being diagnosed genetically during infertility evaluations [19]. Similarly, cases with 47,X,i(X)(q10),Y karyotypes do not express severe phenotypes. Therefore, the cytogenetic screening of these individuals becomes important. Also, follow-up care should be conducted as per the European Academy of andrology guidelines on KS [20]. This guideline provides recommendations and suggestions for care for patients with KS in various developmental stages ranging from childhood and adolescence to adulthood.

## Conclusions

In conclusion, variant KFS cases with mild phenotypes are often missed during childhood and are only diagnosed during puberty, leading to delays in management. Therefore, early genetic analysis, such as karyotyping, becomes important in individuals with testicular abnormalities or hypergonadotropic hypogonadism for the timely management of complications such as infertility and gynecomastia. Also, this will help in premarital counseling for such individuals.
